# Characterization of EEG Data Revealing Relationships With Cognitive and Motor Symptoms in Parkinson's Disease: A Systematic Review

**DOI:** 10.3389/fnagi.2020.587396

**Published:** 2020-11-10

**Authors:** Qing Wang, Lin Meng, Jun Pang, Xiaodong Zhu, Dong Ming

**Affiliations:** ^1^Academy of Medical Engineering and Translational Medicine, Tianjin University, Tianjin, China; ^2^Department of Neurology, Tianjin Medical University General Hospital, Tianjin, China

**Keywords:** Parkinson's disease (PD), electroencephalogram (EEG), motor disorders, cognitive decline, freezing of gait (FOG), dementia

## Abstract

Recent research regards the electroencephalogram (EEG) as a promising method to study real-time brain dynamic changes in patients with Parkinson's disease (PD), but a deeper understanding is needed to discern coincident pathophysiology, patterns of changes, and diagnosis. This review summarized recent research on EEG characterization related to the cognitive and motor functions in PD patients and discussed its potential to be used as diagnostic biomarkers. Thirty papers out of 220 published from 2010 to 2020 were reviewed. Movement abnormalities and cognitive decline are related to changes in EEG spectrum and event-related potentials (ERPs) during typical oddball paradigms and/or combined motor tasks. Abnormalities in β and δ frequency bands are, respectively the main manifestation of dyskinesia and cognitive decline in PD. The review showed that PD patients have noteworthy changes in specific EEG characterizations, however, the underlying mechanism of the interrelation between gait and cognitive is still unclear. Understanding the specific nature of the relationship is essential for development of novel invasive clinical diagnostic and therapeutic methods.

## Introduction

Parkinson's disease (PD) is the second most common neurodegenerative disease after Alzheimer's disease (AD) (Galvez et al., 2018). The degeneration of the basal nucleus nigrostriatal pathway leads to the loss of the dopaminergic neurons, which will result in multiple motor and non-motor symptoms (Martinez-Martin et al., 2011; Galvez et al., 2018). The motor symptoms mainly appear as resting tremor, postural instability, or gait impairment whilst patients would also demonstrate non-motor symptoms, such as depression, anxiety, sleep disorders, and cognitive dysfunctions (Latreille et al., 2015; Galvez et al., 2018). These consequential features of PD contribute to reduced quality of life and increased the risk of disability and mortality in patients with PD.

Gait is no longer regarded as an automated activity, and instead, the role of central nervous system (CNS) and the interaction between motion control and movement execution is drawn more and more attention. Studies in recent decades has revealed a relationship between cognitive dysfunction and motor symptoms of postural instability/gait disability (PIGD) (Martinez-Martin, 2011). Evidence have shown that cognitive functions, especially execution function and attention function, play significant roles in gait regulation (Maruyama and Yanagisawa, 2006; Amboni et al., 2008). Although the precise mechanism underlying PIGD, especially for severe symptoms, such as freezing of gait (FOG), are not completely understood. It has been shown that impaired cognitive functions can reduce motor autonomy of PD patients (Lewis and Barker, 2009), which comprises postural and gait stability (Robinovitch et al., 2013). Such cognitive dysfunctions (i.e., executive function impairment) are significantly related to motor symptoms (Maruyama and Yanagisawa, 2006; Amboni et al., 2008), suggesting that it can be considered as a predictor of PD.

Neuroimaging technologies have been widely used to study the regulation effect of cognition on motor control. Previous research has demonstrated that FOG might be related with dysfunction of the cortical frontal and parietal regions, and increased activation of the frontal region related to attention network has been observed in PD patients (Rushworth et al., 2002; Wager et al., 2004; Peterson et al., 2014; Maidan et al., 2016b). However, current studies have certain limitations as they were mostly performed in a constrained environment due to the immobility of scanner equipment.

Recent mobile brain/body imaging techniques based on electroencephalogram (EEG) allow the acquisition and real-time analysis of brain dynamics during active unrestrained movements (Nathan and Contreras-Vidal, 2016). The approach has been applied to distinguish brain dysfunctions of PD patients (Handojoseno et al., 2018; Wagner et al., 2019). In general, PD patients show a slowing tendency of global EEG activity where a decrease of beta and gamma power whilst an increase of power in the theta and alpha bands have been observed to be significantly related to cognitive processes, such as motivation, emotion, and decision-making (Handojoseno et al., 2013). Event-related potentials (ERPs) do not only reflect cognitive decline but also could indicate motor disorders in PD (Georgiev et al., 2015; Butler et al., 2017; Delval et al., 2018; Maidan et al., 2019).

Despite a certain amount of studies about characterizing EEG patterns in PD have been published, changes of EEG features related to cognition and motor dysfunctions have not been discussed in detail. Therefore, the aim of this article is to provide a literature review of characterizations of EEG signals related to PD's cognitive and motor functions and discuss its potential to be used as a novel method in clinic diagnosis.

## Methods

This review follows a PICO search strategy based on the research question about the brain mechanism related to cognition and motor dysfunctions in PD. The EEG features of PD patients were summarized and compared to healthy controls, as well as other neurodegenerative diseases, such as DLB and AD.

In this review, PubMed, Web of Science databases and EMBASE were systematically searched for relevant literature from 2010 to 2020. Three sets of keywords were used for the literature search: (i) “Parkinson's disease”; (ii) “EEG” or “Electroencephalography”; (iii) “Dual task” or “cognition and motor” or “cognitive and motor.” Papers that have the term of keywords (i & ii & iii) located within the title and/or abstract were included in this review. In addition to the systematic electronic database search, a targeted search of bibliographies of relevant articles was also performed to identify any additional papers for inclusion.

Only original, full-text articles published in English between January 2010 and June 2020 that investigate the cognitive and motor impairments with scalp EEG analysis in patients with PD were considered in this review. Articles were excluded if they: (i) did not use scalp EEG signals to study features associated with cognitive and/or motor symptoms; (ii) studied on other neurological diseases, such as Alzheimer's disease, multiple system atrophy, Huntington's disease, and Binswanger disease; (iii) were review articles.

## Results

### Search Results

**Figure 1:**
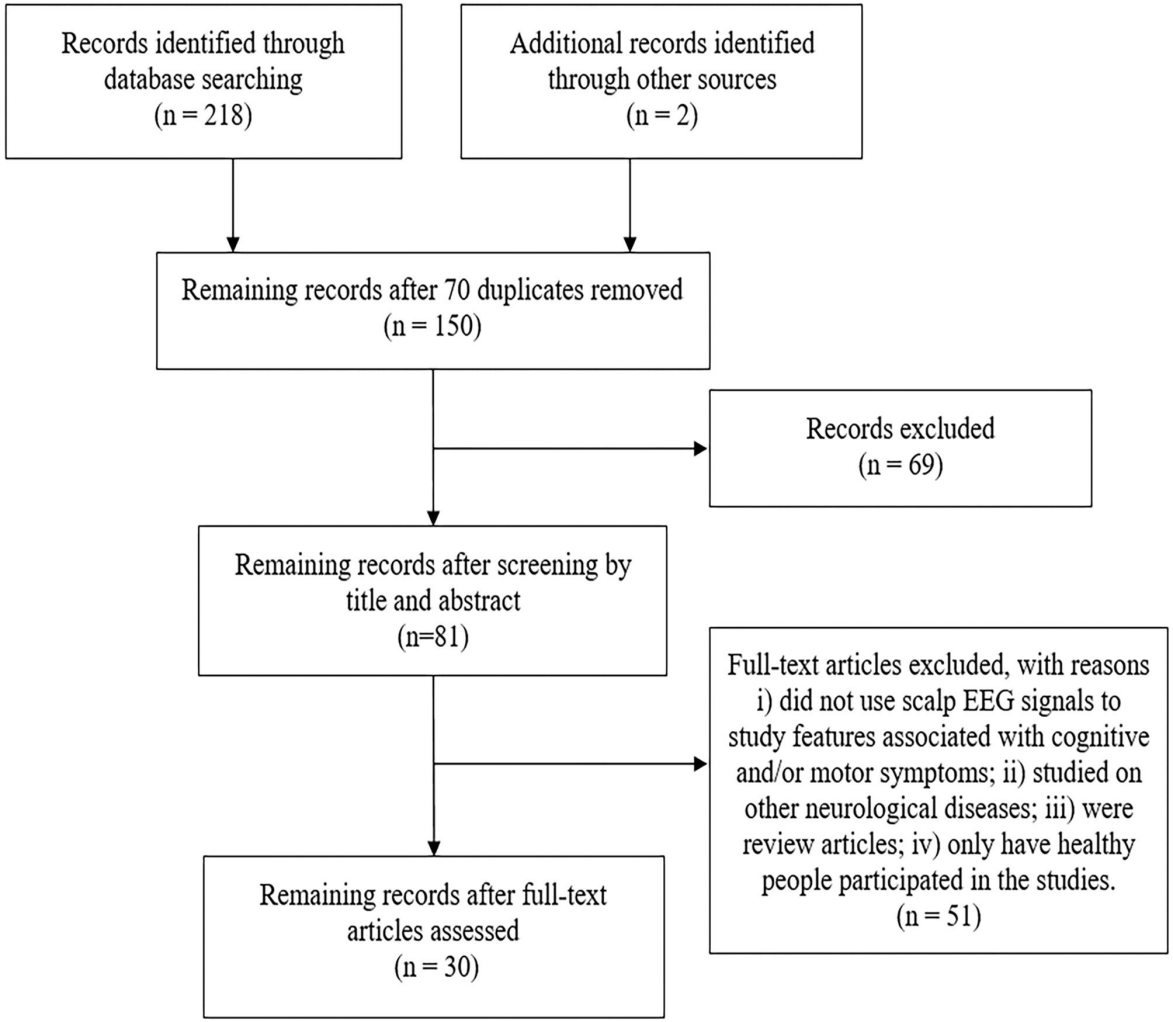
The modified PRISMA flow diagram through the selection of studies.

Using the search method mentioned above, a total of 152 articles were retrieved in the Web of Science database and 66 articles were retrieved in the PubMed database. After duplicate and unrelated papers were removed after initial screening, a total of 51 articles were excluded according to the criteria, of which, 38 were excluded according to the exclusion criteria (i), 8 according to the criteria (ii), 2 according to the criteria (iii) and 3 due to only healthy people participated in the studies. Two relevant articles were identified based on the targeted search in the review article. Therefore, a total of 30 articles were selected for inclusion in this review. A modified PRISMA diagram as shown in Figure 1 illustrates the screening and inclusion process.

### Participants

Fourteen papers enrolled only PD patients with and/or without motor or cognitive impairment in the experiments while the rest of studies aimed to identify difference in EEG features during cognitive and/or motion tasks for PD group compared with healthy control (HC) group (*n* = 14) or patient group with other neurodegenerative disease (*n* = 2), such as AD or Lewy body dementia (LBD). The PD patient demographic characteristics and clinical scores are summarized in Table 1.

**Table 1:** PD patient demographic characteristics and clinical scores (Mean ± SD) are summarized.

**References**	**Number**	**Age (year)**	**Gender (male)**	**Disease duration (year)**	**H&Y stage**	**UPDRS-III**	**Cognition score**
Palmer et al. (2010)	7	63.7 ± 7.1	5	7.1 ± 2.8	–	–	–
Babiloni et al. (2011)	13	72 ± 0	4	–	–	–	17.2 ± 0^b^
Schlede et al. (2011)	19	70.7 ± 0	15	–	–	30.0 ± 0	28.0 ± 0^b^
Bliwise et al. (2014)	64	63.0 ± 9.7	42	5.6 ± 4.0	–	17.6 ± 8.9	28.6 ± 1.7^b^
Herz et al. (2014)	11	60.5 ± 9.4	8	8 ± 5.2	–	17.9 ± 10.9	–
van Wouwe et al. (2014)	10	63.1 ± 0	6	3.52 ± 0	–	13.2 ± 0	29.2 ± 0^b^
Yuvaraj et al. (2014)	20	59.0 ± 5.6	10	5.8 ± 3.5	2.3 ± 0.6	17.1 ± 3.2	26.9 ± 1.5^b^
Caviness et al. (2015)	71	73.7 ± 8.0	43	8.5 ± 4.9	2.27 ± 0.71	22 ± 12	27.8 ± 2.0^b^
Georgiev et al. (2015)	14	60.4 ± 12.3	–	3.5 ± 3.0	1.8 ± 0.4	28.6 ± 7.7	27.6 ± 1.6^a^
Kotz and Gunter (2015)	1	58	1	14	3–4	47	–
Latreille et al. (2015)	68	64.9 ± 0	46	4.5 ± 0	2.3 ± 0	22.5 ± 0	–
Muente et al. (2015)	12	66.5 ± 8.9	5	10.4 ± 6.8	–	22.3 ± 12.9	–
Melloni et al. (2015)	14	56.0 ± 11.2	7	22.0 ± 12.4	6.5 ± 3.5	2.4 ± 0.7	–
Solis-Vivanco et al. (2011)	55	58.5 ± 8.6	33	5.2 ± 3.4	–	–	26.7 ± 2.1^b^
Waechter et al. (2015)	16	64.7 ± 6.2	11	8.9 ± 7.0	2 ± 0.4	30.1 ± 14.8	25.4 ± 2.7^a^
Caviness et al. (2016)	134	75.9 ± 8.2	–	11.4 ± 6.7	2.6 ± 0.9	27.8 ± 14.8	–
Cozac et al. (2016)	37	67 ± 0	25	8 ± 0	–	14 ± 0	–
Quynh Tran et al. (2016)	4	–	3	–	–	–	–
Tard et al. (2016)	25	61.3 ± 8.2	–	7.1 ± 3.1	2.3 ± 0.7	18.0 ± 8.9	27.7 ± 2.2^b^
Utianski et al. (2016)	88	76.4 ± 7.5	–	11.8 ± 6.8	2.6 ± 1.0	27.3 ± 14.4	–
Arnaldi et al. (2017)	54	68.6 ± 7.2	30	13.6 ± 9.9	1.6 ± 0.5	13.4 ± 5.8	28.9 ± 1.1^b^
Butler et al. (2017)	20	63.9 ± 7.8	12	10.25 ± 9.9	2.5 ± 0.4	28.7 ± 12.0	25.2 ± 2.9^a^
Galvez et al. (2018)	14	62.0 ± 6.1	6	7.2 ± 4.9	2.1 ± 0.7	–	–
Handojoseno et al. (2018)	16	70.9 ± 6.9	16	8.6 ± 6.6	2.8 ± 0.6	42.5 ± 14.3	–
Singh et al. (2018)	28	69.8 ± 8.6	17	12.5 ± 3.8	–	22.1 ± 10.1	28.6 ± 1.1^b^
Yoshida et al. (2018)	9	62 ± 6.2	9	10.1 ± 5.2	–	17.6 ± 6.2	26.9 ± 1.7^a^
Babiloni et al. (2019)	120	72.1 ± 0	52	–	–	–	24.4 ± 0^b^
Maidan et al. (2019)	10	60.5 ± 0	6	2.9 ± 0	–	20.2 ± 0	25.2 ± 0^a^
Possti et al. (2020)	6	57.7 ± 0	5	2.9 ± 0	–	21.8 ± 0	25.8 ± 0^a^
Singh et al. (2020)	26	67.4 ± 0	17	6.2 ± 0	2 ± 0	14.9 ± 0	23.3 ± 0^a^

*SD = 0 means that SD value was not provided in the article. SD, standard deviation*.

a *The Montreal Cognitive Assessment (MoCA) scale was used for cognitive level evaluation*.

b *The Mini-Mental State Examination (MMSE) scale was used for cognitive level evaluation*.

### Assessment of Cognitive and Motor Functions

More than half of the studies utilized motor and/or cognitive tasks, some of which combined oddball paradigm (Kotz and Gunter, 2015; Muente et al., 2015; Waechter et al., 2015; Tard et al., 2016; Possti et al., 2020) in order to analyze dual task execution difficulty and investigate the interrelation of the cognitive decline and motor impairment in PD patients. Motor tasks include the standardized Timed up and Go Test (Quynh Tran et al., 2016; Handojoseno et al., 2018), bilateral cyclical ankle movements (Yoshida et al., 2018), lower-limb pedaling motor task (Singh et al., 2020), and repetitive movements of a specific finger (Herz et al., 2014), while the common Simon task (van Wouwe et al., 2014; Singh et al., 2018), and other customized tasks that require more cognitive processing (Palmer et al., 2010; Yuvaraj et al., 2014; Melloni et al., 2015; Solis-Vivanco et al., 2015) were used to accomplish the cognitive tasks. Three other studies established simple auditory and/or visual oddball paradigms to assess PD cognitive and motor symptoms (Georgiev et al., 2015; Butler et al., 2017; Maidan et al., 2019). The characterizations of EEG signals include: EEG frequency spectrum characteristics, evoked potentials and cortical-muscle coherence (CMC). The remaining 11 papers (37%) used resting state EEG to investigate possible biomarkers for cognitive decline in PD patients (Schlede et al., 2011; Caviness et al., 2015, 2016; Latreille et al., 2015; Cozac et al., 2016; Utianski et al., 2016; Arnaldi et al., 2017), evaluate therapeutic effects (Galvez et al., 2018; Babiloni et al., 2019), and distinguish PDD patients from other dementia patients, such as AD and DLB (Babiloni et al., 2011; Bliwise et al., 2014). The EEG frequency spectrum characteristics (the spectral power and/or power density), the event-related desynchronization/synchronization (ERD/ERS), and the EEG connectivity were studied in rest state EEG while sleep EEG, microstate analysis and the grand total EEG (GTE) score were also investigated in PD patients.

## Discussion

This study has evaluated the temporal and spectral characteristics of EEG as well as cortico-muscular coherence (CMC) in PD patients. The definable slowing EEG activity can reflect decreased cognitive level of patients with PD while negative correlation between the GTE score and cognitive state was observed. Reduced ERPs represent the cognitive decline and attention deficits that can be associated with severity of motor symptoms. Abnormalities in β and δ frequency bands are the main manifestation of dyskinesia and cognitive decline in Parkinson's disease, respectively.

### EEG Spectral Characteristics

EEG spectral pattern is usually characterized in the range of 0–30 Hz with five internationally agreed frequency bands: δ (0–4 Hz), θ (5–7 Hz), α (8–13 Hz), β (14–30 Hz), and γ (>30 Hz) bands. It is one of the most studied EEG features in PD patients. Studies showed that cognitive deficits are correlated with a slowing of EEG frequencies. Compared to non-demented PD patients, PD patients with dementia (PDD) have increased amplitude in lower frequency bands (i.e., θ and δ bands) and decreased amplitude in higher frequency bands (i.e., α and β bands; Soikkeli et al., 1991; Stoffers et al., 2007; Caviness et al., 2015; Arnaldi et al., 2017; Geraedts et al., 2018). More studies revealed the increased activities in θ and δ bands of EEG in PD patients (Babiloni et al., 2011; Caviness et al., 2016). Cozac et al. (2016) analyzed low frequency bands and demonstrated that global relative median power (GRMP) spectra of θ band can be used to predict cognitive decline. Despite the attenuated mid-frontal θ activity associated with disease duration, PD patients exhibited increased central θ power and decreased occipital θ power (Palmer et al., 2010; Singh et al., 2018). The abnormality of posterior cortical θ rhythm based on cortical source mapping of resting state EEG was observed in Babiloni et al. (2011). The θ relative band power of PD patients with mild cognitive impairment (PD-MCI) was higher than that of cognitively normal PD patients (PD-CogNl). An increasing trend was observed in the δ band power from PD-CogNl to PD-MCI to PDD (Caviness et al., 2016). Longitudinal changes of neuropsychological test were mostly related to the change of δ power for PD patients with mild to moderate motor impairment (Caviness et al., 2015). PD patients had higher diffuse δ source compared to HC group which may lead to abnormal sources of central δ rhythms in PDD patients (Babiloni et al., 2011, 2019). The increased power in δ band could be the main feature of the deterioration of cognitive impairment, which is closely related to the incidence of PDD (Caviness et al., 2015, 2016).

Event-related desynchronization (ERD) and event-related synchronization (ERS) are considered to indicate the activation of the motor cortex during planning, executing and completing a movement (Heinrichs-Graham et al., 2014; Tard et al., 2016; Delval et al., 2018). Previous studies have shown the α band rhythm display an essential role in motor processing and enhanced β band oscillation may reflect motor control deficits during movement preparation (Cahn et al., 2013; Tard et al., 2016; Delval et al., 2018). The diminished β power ERD and prolonged β ERS in an attentional experiment suggest that the coupling between attention and motor preparation was impaired in PD-FOG (Tard et al., 2016). The relative bandpower in α band showed a decreasing trend in the longitudinal changes from PD-CogNl to PD-MCI to PDD as well as cortical changes were observed in PD patients (Babiloni et al., 2011, 2019; Caviness et al., 2016). PD patients had lower occipital α source compared to HC whilst PDD patients showed increased frontal-temporal α source and abnormal sources of posterior cortical β1 rhythm (Soikkeli et al., 1991; Babiloni et al., 2011, 2019). As the changes of β band power are related to gait initiation failure (GIF) events, abnormal β oscillation may be a biomarker of motor impairment in PD patients (Palmer et al., 2010; Quynh Tran et al., 2016).

The efficient neural communication between premotor and motor cortical areas was critical for motor control (Herz et al., 2014). The decrease in γ band power was observed in resting state EEG of PD patients (Stoffers et al., 2007). PD patients exhibited a loss of γ-γ coupling from the lateral premotor cortex (lPM) to the supplementary motor area (SMA) when performing repetitive movements of the right index finger at maximal rate. Cross-frequency coupling of the slow and fast band activities might facilitate information transmission across brain networks (Canolty and Knight, 2010). Kappa_w_ was used to evaluate the weighted brain network and found the parameters increased in the θ and β bands while decreased in the δ and α bands in PD patients compared to healthy participants (Utianski et al., 2016). The results showed a hub shifting in the bands and peak background frequency exists in both higher and lower frequency bands. The shift from α/δ to θ/β coupling suggests a change in cortical network in PD (Utianski et al., 2016), and the enhancement of θ/β coupling from primary motor cortex (M1) to the lateral premotor cortex (LPM) is related to the improvement of levodopa-induced motor function (Herz et al., 2014). The brain graph theory network analysis based on EEG signals indicated that the network breakdown is associated with cognitive decline, manifested as less connectivity and decreased global network efficiency with a loss of highly connected cortical hubs (Utianski et al., 2016).

Changes of EEG spectrum can be associated with clinical assessment scales and motion performance. The power of θ and β bands are, respectively related to the scores of Montreal Cognitive Assessment (MoCA) and the motor part of the Unified Parkinson's Disease Rating Scale (UPDRS-III) (Singh et al., 2020). PD-FOG patients showed increased β power and decreased θ power in mid-frontal region when performing a lower-limb pedaling motor task needing intentional initiation and stopping of movement (Singh et al., 2020). The θ, α, and β activities in left posterior parietal cortex (PPC) were positively correlated to the motion parameters during single or dual task walking (conversation/email sending), indicating that the left PPC may be involved in sensory motor integration and gait control (Pizzamiglio et al., 2018). Studies also found that the power of δ, θ, and γ bands was significantly increased during the planning and execution stages of motor (particularly in the execution stage), followed by decreases in α, β, and low-γ bands after an execution cue (Combrisson et al., 2017; Delval et al., 2018).

**Figure 2:**
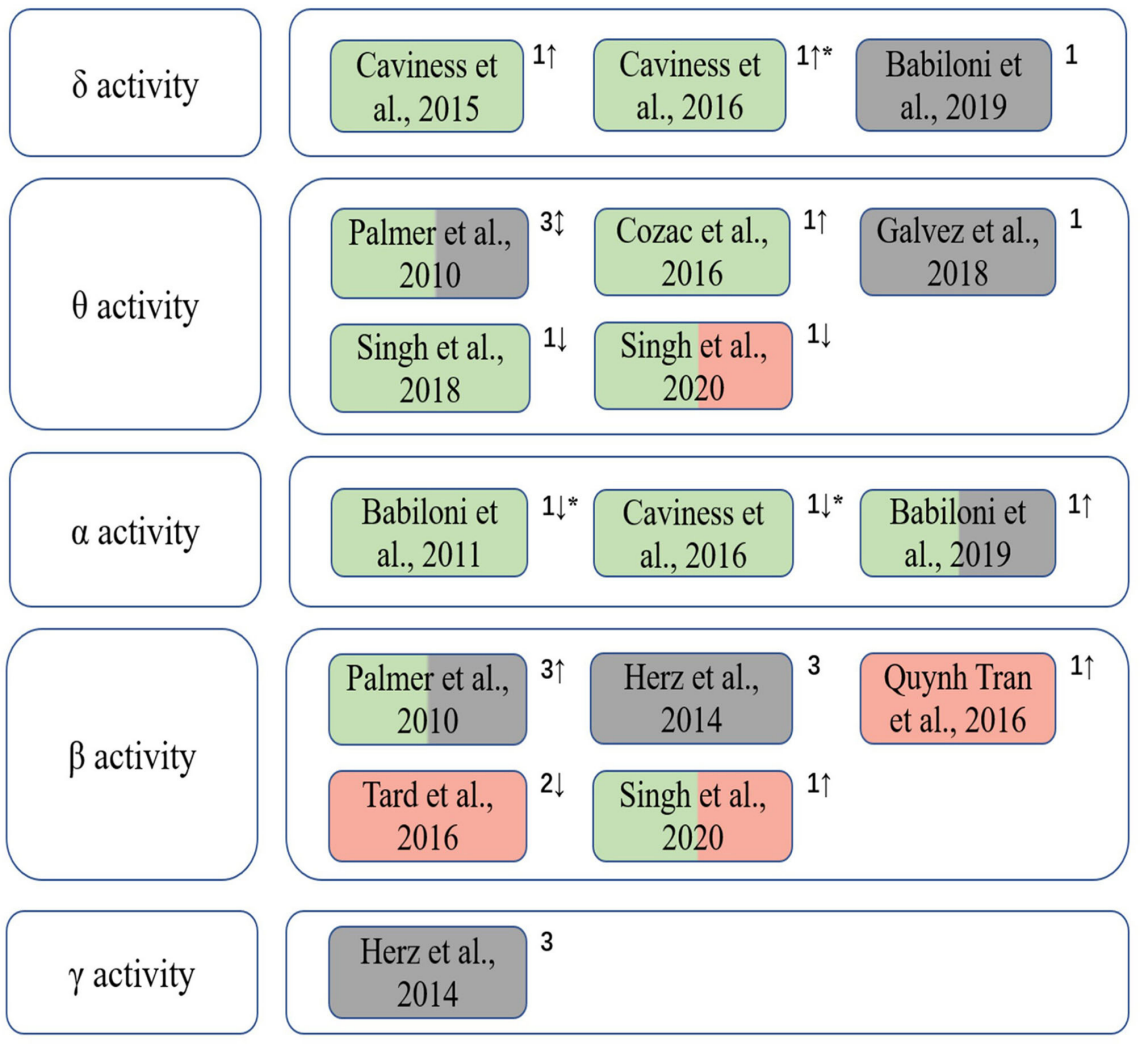
The correlation between EEG spectral characteristics in different frequency bands and cognition and motor functions of PD patients. Green indicates that the EEG spectral characteristics is correlated with cognitive function; red indicates that it is correlated with motor performance; gray indicates that EEG spectral patterns are used to assess clinical treatment outcomes. 1, spectral power/power density; 2, event-related desynchronization/synchronization (ERD/ERS); 3, EEG connectivity; “↑,” the increase trend; “↓,” the decrease trend; “↕,” different trends of EEG spectral characteristics in various brain regions when cognitive and/or motor symptoms appear; “*” indicates that the gradual changes of EEG features of patients from PD-CogNl to PD-MCI to PDD manifest decrements in cognitive level.

Figure 2 summarizes the included studies on EEG spectrum characteristics related to Parkinson's disease in this review. A significant interrelation was observed between β band power changes and dyskinesias, whilst abnormalities in θ and δ bands were associated to cognitive decline in PD patients. The changes of the spectrum characteristics can be used to assess the cognitive and motor abilities of PD patients during treatments as a dynamic and quantitative method.

### Event-Related Potentials (ERPs)

Event-related potentials (ERPs) refer to event-related voltage changes in brain regions in response to specific stimuli (e.g., visual, auditory, and somatosensory stimuli). The ERPs provide a powerful method for exploring the ongoing EEG activity that are time-locked to sensory, motor, and cognitive events, and can be used as an electrophysiological indicator of cognitive function (Butler et al., 2017; Maidan et al., 2019). Table 2 summarizes ERP-related studies included in this review. The results demonstrate that changes of ERPs have been utilized to identify cognitive decline and abnormal attention distribution of PD patients, and can be applied to investigate the correlation between cognitive and motor functions. All studies investigated ERPs induced by visual or auditory stimuli (*n* = 10), and five studies also discussed event-related potentials while performing motor tasks (*n* = 5).

**Table 2:** Summary of experimental paradigms and main results of induced ERPs.

**Induced method**	**ERPs induced**	**References**	**Subjects**	**Experimental paradigm**	**Main results**
Visual/auditory stimulations only	P300	Georgiev et al., 2015	14 PD patients, 13 HC	Auditory three stimuli Oddball paradigm: 500 and 1,000 Hz, white noise. Visual Oddball paradigm: a small light blue circle at 4.36°, circle at 5.72°, checkerboard pattern. Subjects were asked to count the times the target stimulus appeared.	Dopaminergic drugs significantly increase the P3a amplitude in PD patients, but only in response to error select stimuli in hearing rather than vision.
		Muente et al., 2015	12 PD patients, 12 HC	Auditory Oddball paradigm: 635 and 435 Hz. Subjects should press the key of 1–9 to form RNG/ONG when hearing the standard stimulus, and press the 0 key when hearing the target.	Compared with ONG, P3b amplitude in PD patients is significantly smaller when generating RNG. But in HC group, it is similar in two conditions.
	P300, MMN, RON	Solis-Vivanco et al., 2015	55 PD patients, 24 HC	Auditory experiment: 1,000 Hz (90%), 1,100 Hz (10%). Subjects are asked to distinguish the sound and press the corresponding button at full speed.	Both the duration and severity of PD have important effects on P3a amplitude. The amplitude of P3a in PD is significantly lower than that in HC, especially in patients with H&Y stage 2 and 3.
	P600	Kotz and Gunter, 2015	1 advanced PD patient	Auditory Oddball paradigm: 600 and 660 Hz. Subjects should count the number of target stimuli. They then receive a language part in which passively listening to grammatically correct and incorrect sentences. Sections 2 and 3 give subjects auditory stimulus of progress/waltz before the language part while section 1 and 4 not.	External auditory cues seem to completely restore the P600 potential of PD patients in response to a syntactic expectancy violation, such as March (4/4).
	LRP, P300	Butler et al., 2017	10 PD-FOG, 10 PD-nFOG	Visual Oddball paradigm: a vertical green cross blinking in the screen, cross rotating 45°. Subjects were asked to press the button immediately after the target.	The LRP in PD-FOG appears earlier and has a larger amplitude. The LRP amplitude is significantly correlated with the FAB score, but not with the disease severity indicators such as H & Y and UPDRS-III scores.
Merging motor tasks	P300	Waechter et al., 2015	9 PD-FOG, 7 PD-nFOG	VR technology provides a virtual corridor which subjects walk through. They complete the visual Oddball task at the same time: “+,” “×.” Subjects are required to press the button when seeing target. Finally, repeating the above experiment in a sitting position.	The P3b component in walking/sitting experiments could not be detected in PD-FOG if the data is stimulus-locked, while P3b is clearly visible when respond-locked (lock to the button press).
		Maidan et al., 2019	11 healthy young adults, 10 healthy older adults, 10 PD patients	Auditory Oddball paradigm while standing or walking on the treadmill: 600 Hz pure tone bursts, 1,200 Hz. Subjects need to count how many times the target stimulus appeared.	The P300 latency of the elderly and PD patients is prolonged during walking, but the P300 amplitude only reduces in PD patients; better motor and cognitive abilities are associated with shorter P300 latency.
	N1, P2, P3	Tard et al., 2016	12 PD-FOG, 13 PD-nFOG, 13 HC	Auditory Oddball paradigm: 1,000 and 2,000 Hz. Subjects need to press the button after the target stimulus. Simultaneously performing a motor preparation task: taking right foot to start walking as soon as the screen shows “go.”	The ERP characteristics of three groups are not significantly different. As a post-perceptual marker of FOG, EEG oscillations are more sensitive than ERP.

*LRP, lateralized readiness potential; SDN, step-cue delay negative; RNG, random number generation; ONG, ordered number generation; MRP, movement-related brain potential; CPP, centroparietal positivity; APA, anticipatory postural adjustments; VR, virtual reality; H&Y, Hoehn and Yahr scale; UPDRS III, the motor part of the Unified Parkinson's Disease Rating Scale*.

#### Auditory and/or Visual ERPs

**Figure 3:**
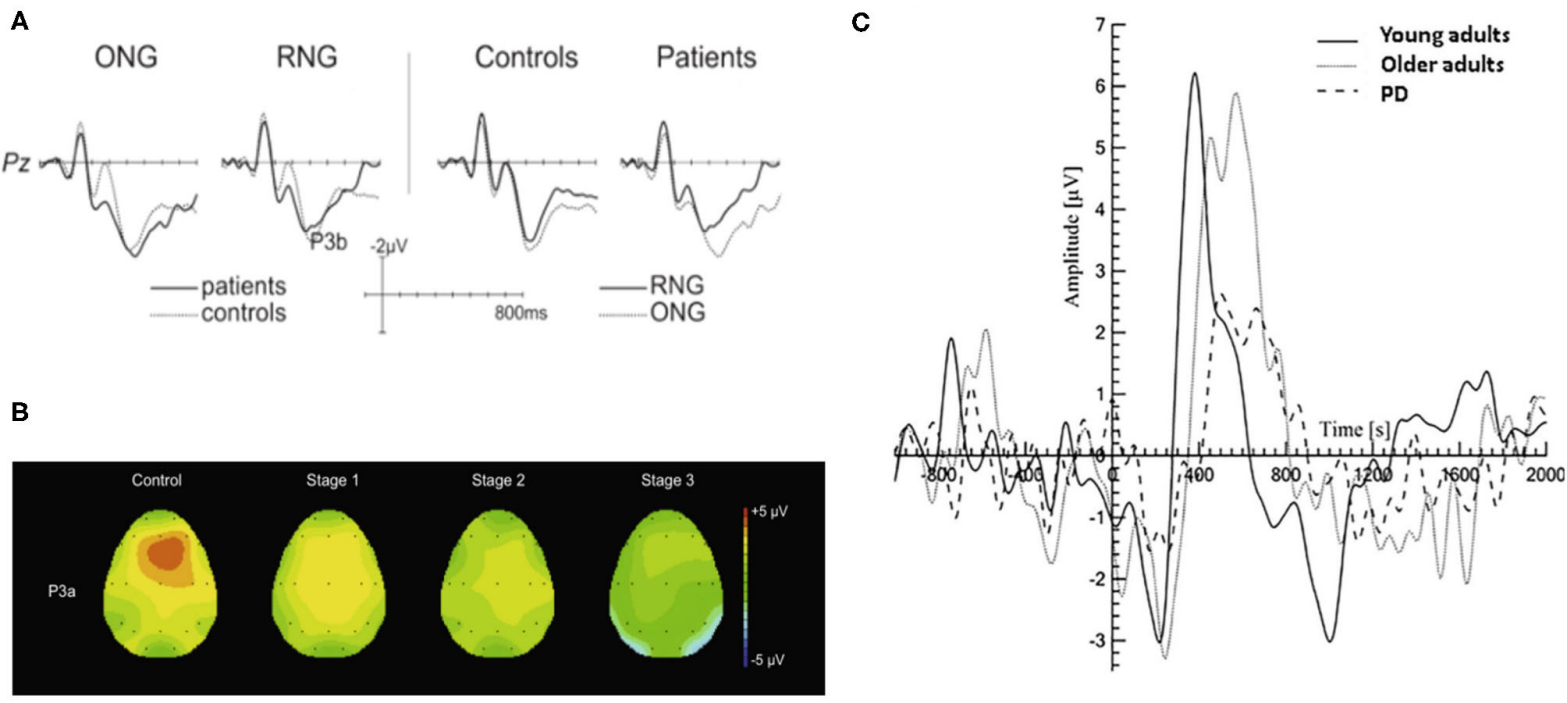
Typical P300 changes in PD patients. **(A)** A more pronounced reduction in P3b amplitude during the RNG task was observed than that with ONG task (Muente et al., 2015). **(B)** Topographic maps of P3a in HC group and PD patients in three H&Y stages (stage 1, 2, 3) during an auditory oddball paradigm. The P3a showed a frontocentral distribution and its amplitude was negatively associated with the severity of PD (Solis-Vivanco et al., 2015). **(C)** The P300 amplitude is significantly smaller in PD patients (dash line) compared to those in young and older adults (solid black line and gray line, respectively) during walking (Maidan et al., 2019). HC, healthy control; H&Y, Hoehn and Yahr scale; RNG, random number generation; ONG, ordered number generation. (Image sources were adapted with granted copyright permission.) Figure 3A is available at IOS Press through Muente et al. (2015).

The P300 ERP is the most investigated neural markers of attention and cognition. Studies have shown that changes in the amplitude and latency of the P300 ERP are related to attention and cognitive decline (Georgiev et al., 2015; Maidan et al., 2019). An increase of P3b latency was observed in PD patients in visual and/or auditory oddball paradigms and associated with disease severity, cognitive dysfunction and impaired activities (Matsui et al., 2007; Da Silva Lopes et al., 2014). It is shown in Figure 3A that PD patients had a significantly smaller amplitude of P3b in random number generation (RNG) task compared to that in ordered number generation (ONG) task. The execution of RNG task may require greater attention resources, therefore less random behavior in PD patients was observed due to depleted attention resources (Muente et al., 2015). As there is a relationship between the decrease of P3a amplitude and the duration and severity of PD, the P3a is regarded as a potential cognitive marker for the development of mild to moderate PD patients (Solis-Vivanco et al., 2015). The changes of EEG in PD patients can be used to identify the drug effect during treatment in which P3a amplitude is mainly related to motor symptoms and dopaminergic medication (Georgiev et al., 2015). However, it should be noted that the P300 ERP is highly sensitive to cognitive decline and attention disorders, which may also occur in other neurodegenerative diseases, such as AD (Maidan et al., 2019). Further study on P300 in a large representative sample will be needed to support the potential use of P300 as a biomarker for PD patients.

Another measure commonly used to study ERPs is Event-Related Spectral Perturbation (ERSP), which evaluated the spectral power within various frequency bands (Possti et al., 2020). Changes in brain dynamics in the power of different frequency bands, i.e., delta, theta, alpha, and beta bands, were observed when PD patients, healthy elder, and young subjects participated in an auditory oddball cognitive task performed during standing and walking on a treadmill (Possti et al., 2020). PD patients showed higher alpha and beta power during single and dual task compared to older adults, while an early lower delta power was also found during dual task. The phenomenon suggests that further modulation of readiness and attention is required in elders and PD patients and leads to larger recruitment of brain resources.

Unexpected changes in task-irrelevant auditory stimuli are usually accompanied by the elicited ERP components associated with attention orientation, such as novelty-P3a, mismatch negativity (MMN), reorientation negativity (RON). They are collectively referred as “distraction potential” and have been used to study the brain mechanism of involuntary attention (IA) in PD patients (Solis-Vivanco et al., 2011). As shown in Figure 3B, Solis-Vivanco et al. (2015) found that PD patients, especially those receiving anti-PD drug treatments, had reduced P3a amplitudes in the frontal, central, and temporal regions during distracting oddball tasks. The changes in novelty detection and/or the direction of attention were only observed in severe PD patients (Karayanidis et al., 1995; Georgiev et al., 2015; Solis-Vivanco et al., 2015). It suggests that the MMN might be potential in identifying patients with PD dementia (PDD) (Bronnick et al., 2010). Solis-Vivanco et al. (2011) also studied the role of RON in assessing cognitive function of PD patients. Comparing with drug-naïve patients, RON amplitude of patients receiving drug treatment was significantly higher and closer to HC group, indicating that dopamine therapy can also regulate the reorientation of attention. It is proven that there is a correlation between RON latency and the behavioral outcome of advanced PD patients (Georgiev et al., 2015; Solis-Vivanco et al., 2015). Despite the above-mentioned potentials, Kotz and Gunter (2015) firstly studied the P600 potential in one patient with advanced PD for reflecting language-related deficits.

#### Movement-Related Potentials

Understanding the neural mechanism underlying the cognitive-motor interference may have implications for predicting the decrement in dual task performance. The P300 ERP is regarded as an index of the amount of resources allocated to stimulus processing. The reduced amplitude of P300 is associated with worse attentional concentration and the longer latency is related to motor and cognitive abnormalities (Polich, 2007; Maidan et al., 2019). PD patients showed a decrease amplitude and an increased peak latency of P300 during sitting and walking tasks as shown in Figure 3C, but its amplitude when performing oddball task during standing is similar to those in healthy elderly and young groups (Yilmaz et al., 2017; Maidan et al., 2019). There is no significant association between FOG symptom and ERPs. The ERPs elicited in auditory oddball paradigm may not be suitable as a biomarker of FOG symptoms (Tard et al., 2016).

The lateralized readiness potential (LRP) reflects the preparation of motor activity and it is modulated by participants' observed response. Due to the lack of motor preparation in PD caused by dysfunctions in supplementary motor area (SMA) (D'Ostilio et al., 2013), the LRP might occur earlier and have larger amplitude in PD-FOG when comparing to PD-nFOG (Butler et al., 2017). The increased activation in the prefrontal cortex area in the occurrence of FOG suggests that PD patients with FOG would need more attentional source (probably from the lateral premotor area) compared with PD-nFOG. The change in LRP reflects over-recruitment of the lateral premotor area in order to compensate for SMA deficits, and it also indicates that the main defect of FOG might occur in the motor preparation stage (Shine et al., 2013). As the LRP can be regarded as a movement-related brain potential, the results from van Wouwe et al. (2014) demonstrated that inhibition of motor cortex is important to alleviate PD symptoms. Another study also found that PD patients had reduced motor potential (MP) and impaired action-sentence compatibility effect (Melloni et al., 2015).

### Other EEG studies in PD

#### EEG Microstates

**Figure 4:**
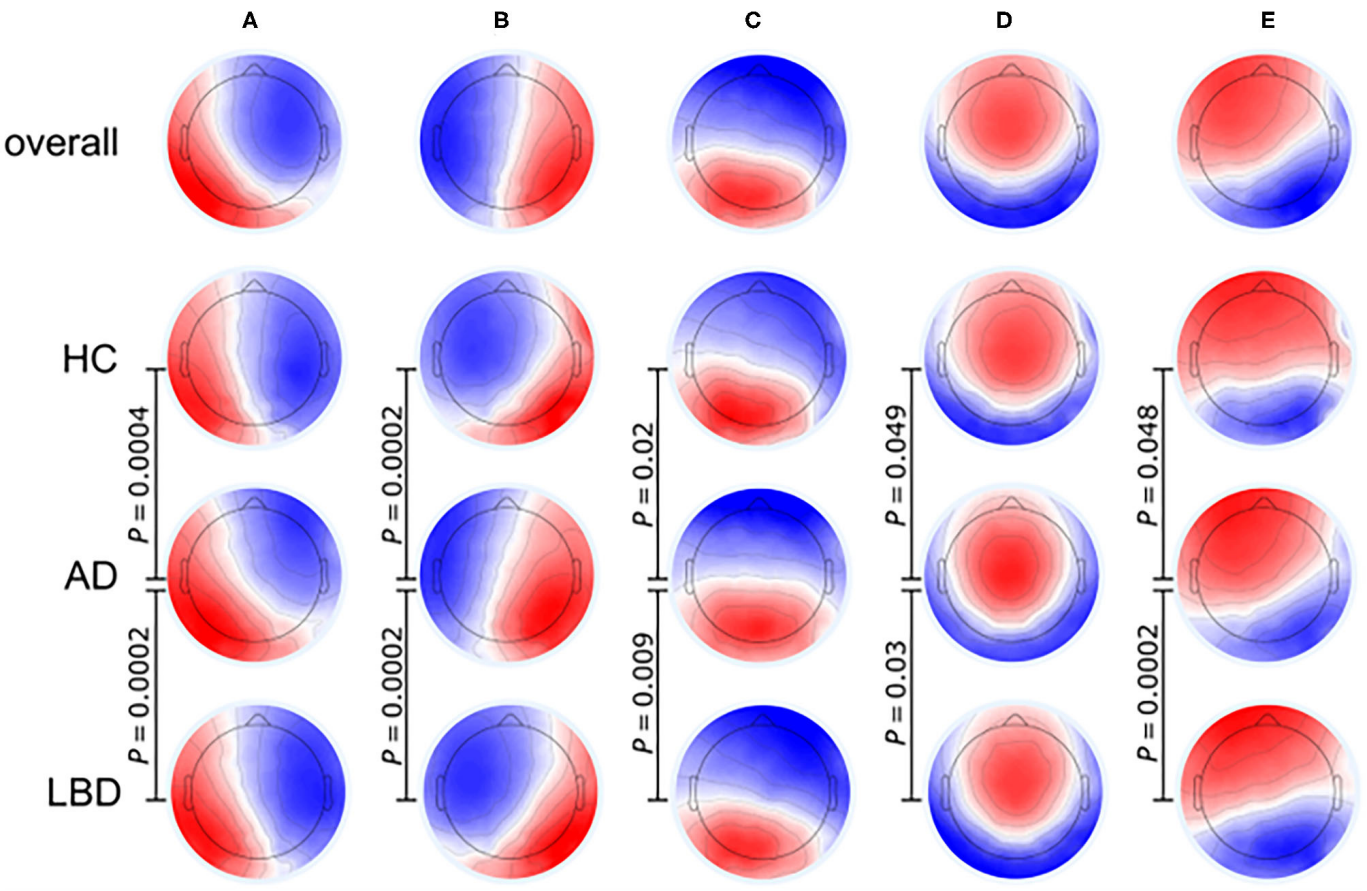
Microstate class topographies for dynamic brain activities in Alzheimer's disease (AD) and Lewy body dementia (LBD) patients compared to healthy control (HC) group. The EEG signal was divided into a group of short, non-overlapping and quasi-stable microstates where the microstates **(A–E)** were related to over 70% of changes in brain activities. *P*-values result from comparing the group topographies between groups using TANOVA. (Image source: Schumacher et al., 2019b: use permitted under the Creative Commons CC BY license).

EEG signals can be segmented into many different short topographies (microstates) which can be clustered into a limited number of landscape (Koenig et al., 1999). EEG microstates reflect a transient and stable brain topology on a millisecond temporal level and provide a powerful perspective on the dynamic changes of the brain. It has been proved that EEG microstate features, especially its duration, were associated with the cognition and perception, and different cognitive functions were related to specific microstates (Britz et al., 2010; Van de Ville et al., 2010; Milz et al., 2016). Figure 4 illustrates that the EEG microstate analysis can be used for investigating brain activity changes in neurodegenerative diseases. Compared to AD and HC groups, patients with Lewy body dementia (LBD) have longer durations of all microstate classes and the number of distinct microstates per second is also reduced (Schumacher et al., 2019b). As the fluctuation of cognitive and attentional disorders is an important feature of Lewy body dementia, the severity of cognitive fluctuation is positively associated with the slowing of microstate dynamics (McKeith et al., 2005, 2017).

The slowing of microstate dynamics indicates a relative loss of brain variability at resting state in patients with LBD (Schumacher et al., 2019b), which is consistent with the loss of brain network flexibility observed in previous fMRI studies (McIntosh et al., 2008; Jia et al., 2014; Schumacher et al., 2019a). The average duration of microstates in LBD patients is inversely related to dynamic functional connections between the basal ganglia and thalamus or large cortical networks, indicating that the dynamic interactions in the cortical-basal ganglia-thalamic loop participate in the regulation of global microstate dynamics and the subcortical abnormalities have a significant impact on the overall function of the whole brain network (Bell and Shine, 2016; Schumacher et al., 2018).

#### Sleep EEG

Recent studies show that sleep spindle and slow waves might facilitate understanding the mechanism of brain plasticity and the results have been related to human cognitive ability. However, the potential use of sleep EEG as a biomarker of cognitive decline in PD is still unclear (Latreille et al., 2015). In a longitudinal study, PD patients who develop dementia after 4.5 years of follow-up study showed decreased density and amplitude of sleep spindle in baseline polysomnography at the posterior cortical area: The PDD patients had most decrease in sleep spindles density, followed by PD patients without dementia, and the HC group has the least (Latreille et al., 2015). The result indicates that the sleep spindle density might be used a potential marker of cognitive decline in PD.

Cognitive fluctuation is a noteworthy feature of DLB rather than PD (Bliwise et al., 2014). In comparison of the coefficients of variation (COVs) based on the polysomnography, DLB patients have significantly greater cognitive fluctuation that PD patients even when they have similar level of cognitive impairment. Although PD patients have smaller amplitude of slow waves compared to healthy people, there is no significant difference observed between patients with different cognitive levels. Slow waves might not be suitable for predicting the cognitive function (Latreille et al., 2015).

#### Grand Total EEG (GTE) Score

The grand total EEG (GTE) score is a rating scale for clinical EEG analysis which can be used for the diagnosis of AD and LBD. It can distinguish between AD and other types of dementia (Pijnenburg et al., 2008). The original GTE score consists of six subscales: (i) frequency of rhythmic background activity, (ii) diffuse slow activity, (iii) reactivity of rhythmic background activity, (iv) paroxysmal activity, (v) focal abnormalities, and (vi) sharp wave activity. A negative relationship between scores (i)–(iii) and cognitive decline was observed in Schlede et al. (2011) by analyzing the correlation between GTE scores and cognitive levels in PD patients. The scores could be potential to identify MCI, which is a common non-motor symptom in PD and regarded as a transitional state between normal cognition and PDD.

#### Cortico-Muscular Coherence (CMC)

The cortico-muscular coherence (CMC) reflects functional coupling between the cortical activity and muscle activity (Wagner et al., 2019). It has been widely used in studies to assess the recovery of motor function after stroke by quantifying interaction between the motor cortex and controlled muscles, however the CMC is rarely used in PD patients (Ushiyama et al., 2011; Zheng et al., 2018).

The findings in CMC features of PD patients are not yet consistent. Several studies have shown that PD patients exhibit motor abnormalities during ankle movement (Yoshida et al., 2018; Zheng et al., 2018). Although there was no difference in CMC even when movement performance significantly varied between PD patients and age-matched HC group (Andrykiewicz et al., 2007). The increased CMC of the midline cortical areas and tibialis anterior muscle in β band during maximum dorsiflexion and plantarflexion is opposite to results from previous study in which decreased β band CMC in PD patients during continuous isometric wrist extension was found (Omlor et al., 2007). Levels of cortico-muscular β band coherence during forearm contraction were found to be similar in patients with PD and HC group (Pollok et al., 2012). Different sensorimotor loops are responsible for upper and lower limb movements, which may explain the inconsistent results among the existing studies. The task-related and movement-related effect of CMC should be concerned when researchers investigate changes of functional interactions between brain activity and movement kinematics using CMC analysis.

### Changes of Brain Networks in PD

**Figure 5:**
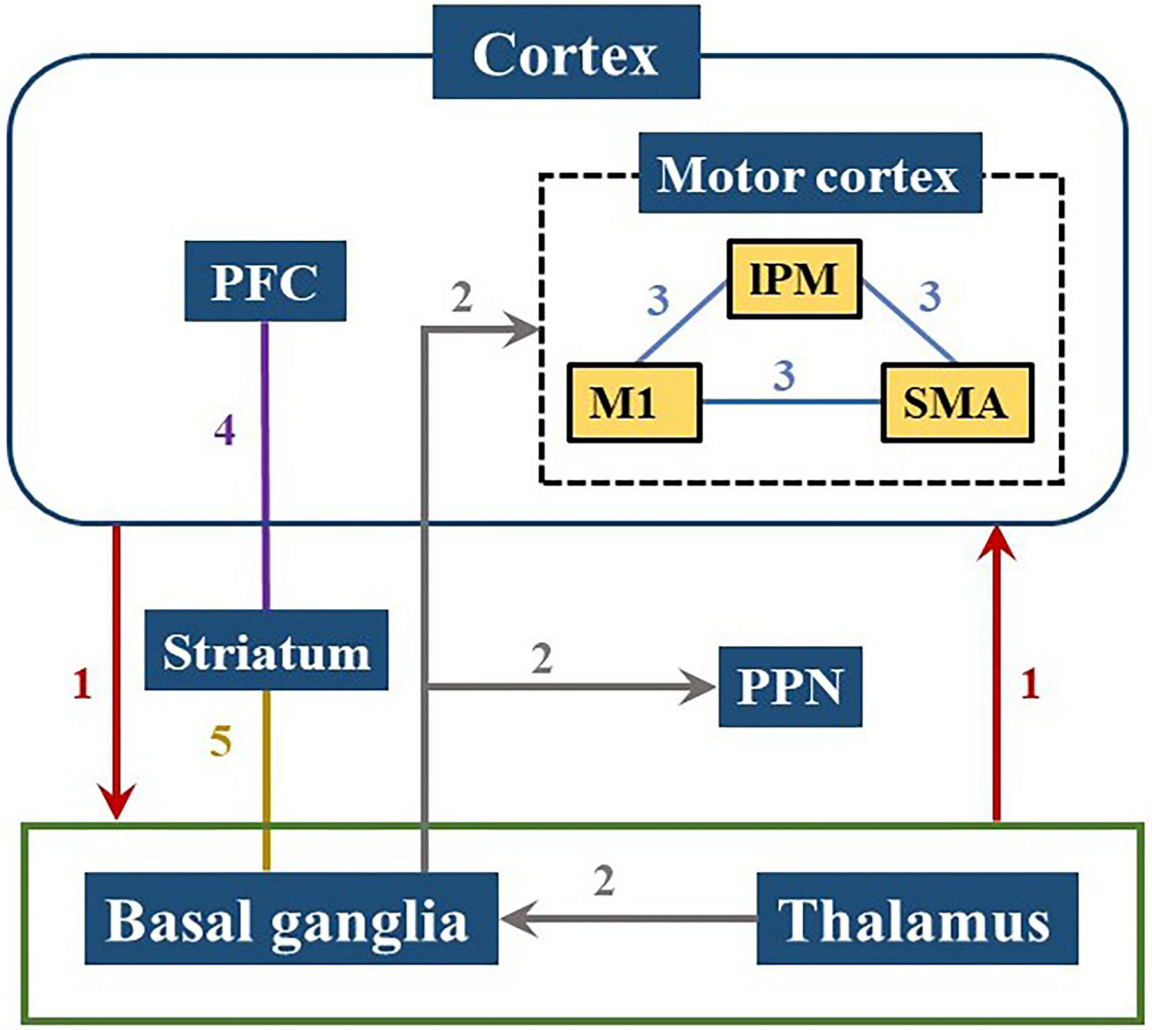
Brain pathways involved in PD cognitive and motor dysfunctions. Neural information is transmitted from the cerebral cortex to the basal ganglia or thalamus and back to the cortex as shown in path 1. Path 2 demonstrates that the input from the thalamus in the basal ganglia is projected to the motor cortex or PPN, reflecting the pathological activities within the ganglia which can affect motor control process. The internal connection of the motor cortex in path 3 has a great influence on motor functions of PD patients. Path 4 and 5 are related to the striatum showing that the frontostriatal circuit and the basal ganglia-striatal circuit are both damaged in PD patients. PFC, prefrontal cortex; lPM, lateral premotor cortex; M1, primary motor cortex; SMA, supplementary motor area; PPN, pedunculopontine nucleus.

PD patients have less global efficiency and functional connectivity of the brain network during the cognitive level declines (Utianski et al., 2016), which might be caused by pathological oscillations and abnormal regulation in the cortico-basal ganglia-thalamic-cortical pathways (Palmer et al., 2010; Herz et al., 2014). Studies have shown that the motor and cognitive dysfunction of PD patients can be related to abnormal changes in multiple pathways involving several brain regions, mainly including the basal ganglia (BG), thalamus, and frontal region. The changes of brain networks in PD were summarized in Figure 5.

The BG circuits play a key role in high-level cognitive function and motor control (Melloni et al., 2015). The loss of dopamine impacts the network between the prefrontal cortex and BG which leads to inability to coordinate the selection and inhibition of conflict responses (Aron et al., 2007). The damage of the BG-cortical motor network consisting the loop from the frontal lobe to BG/thalamus and back to the cortex, may cause downstream influence on the cortical-subcortical connectivity and interfere with the cortical-subcortical motor network (Garcia and Ibanez, 2014; Melloni et al., 2015). Moreover, the cortical-BG-thalamic circuit is an important contributor to large-scale network communication in the brain, in which the dynamic interaction of the various components plays a role in the regulation of brain dynamics (Bell and Shine, 2016). Several different locations in the frontal area are connected to specific areas of the striatum and thalamus through parallel circuits (Cummings, 1995). Pathologic activities in the ganglia are input from the thalamus to the BG, and then projected to the motor cortex or pedunculopontine nucleus (PPN) (Lanciego et al., 2012; Hunnicutt et al., 2014; Yoshida et al., 2018). Dysfunction within these circuits lead to symptomatic progression in PD, such as cognitive issues.

Insufficient motor preparation in PD patients is mainly caused by supplementary motor area (SMA) dysfunction and can be compensated by overrecruiting larger cortical areas, such as lateral premotor cortex (lPM) (D'Ostilio et al., 2013; Butler et al., 2017). Both lPM and SMA are located in Brodmann Area 6 (BA 6), while the primary motor cortex (M1) is in Brodmann Area 4 (BA 4). These three regions are all located near the precentral gyrus and the connections between them (shown as Path 3 in Figure 5) affect movement function in PD (Herz et al., 2014). Increased activities in the prefrontal cortex (PFC) has been observed in PD patients during dual-task walking, which indicates a potential compensation mechanism involving the recruitment of attention networks (Holtzer et al., 2015; Maidan et al., 2016a, 2019). The activation of the PFC has been proven to have an essential role in cognitive control (Singh et al., 2018) and also involve in initiation and execution of movement (Singh et al., 2020).

### Limitations and Perspectives

The EEG analysis provides parameters to quantitatively evaluate cognitive deficits related to the severity of PD, making it a promising approach to assess the therapeutic efficacy. Galvez et al. (2018) investigated the possibility of binaural-rhythmic sound simulation to be applied as an effective therapy using EEG data. The results showed that the decrease of θ power in PD patients after receiving auditory stimuli, demonstrating that the changes of EEG in PD patients could detect the drug effect before and after the treatment (Georgiev et al., 2015). The P3a amplitude is mainly related to motor symptoms and dopaminergic medication. Moreover, the onset of FOG could be identified by tracking the dynamic changes of EEG (Shine et al., 2014; Handojoseno et al., 2015, 2018). Handojoseno et al. (2018) established a classifier with a sensitivity of 85.86% and a specificity of 80.5% for the prediction of the transition period of FOG (5 s before FOG onset).

Studies have shown that EEG energy power, entropy, and signal correlation are significantly related to FOG symptom in PD. However, the relationship between cognitive and motor symptoms in PD is not well-understood. Current studies have a variety of sample size and experimental setting (Herz et al., 2014; Georgiev et al., 2015; Singh et al., 2018), thus leading to inconsistent conclusions. Further studies are needed to elucidate the relative contributions of EEG patterns on reflecting the cognitive and motor symptoms and their relationship. A better understanding of neural mechanism based on EEG analysis related to PD is crucial to develop novel invasive clinical diagnostic and therapeutic methods.

## Conclusion

This paper reviewed recent 10 years studies related to cognitive and motor deficits in PD patients using EEG analysis. The studies show that PD patients have significant changes in specific EEG patterns compared to healthy people. Movement abnormalities and cognitive decline can be related to changes in EEG spectrum and ERPs during typical oddball paradigms and/or combined motor tasks. The unique time-frequency characteristics of EEG could provide further insight into disrupted sensorimotor networks in PD with motor deficits, such as FOG, dyskinesia, and dystonia in a real-world environment. However, the mechanism underlying interaction between gait and cognition still needs to be further studied.

## Data Availability Statement

The original contributions presented in the study are included in the article/supplementary material, further inquiries can be directed to the corresponding author/s.

## Author Contributions

LM, QW, XZ, and DM: conception and design of the study. QW and JP: acquisition, analysis, and interpretation of literatures. QW and LM: drafting the manuscript. All authors critically revised the draft and approved the final version.

## Conflict of Interest

The authors declare that the research was conducted in the absence of any commercial or financial relationships that could be construed as a potential conflict of interest.
